# A study on the current state and equity level of social participation ability among older adults in Henan Province, China

**DOI:** 10.1186/s12877-022-03022-6

**Published:** 2022-04-19

**Authors:** Weicun Ren, Dongming Chen, Clifford Silver Tarimo, Qingfeng Tian, Jian Wu, Yinying Wang, Shangying Li

**Affiliations:** 1 Department of Health Management, Sanquan College of Xinxiang Medical University, Xinxiang, China; 2 College of Public Health, Zhengzhou University, Zhengzhou, China; 3 Department of Science and Laboratory Technology, Dares Salaam Institute of Technology, Dar es Salaam, Tanzania; 4 Medical Security Supervision Office, Luolong District Medical Security Bureau, Luoyang, China

**Keywords:** Older adults, Social participation ability, Equity, Structural Equation Model, Dynamic matter-element analysis

## Abstract

**Background:**

The social participation ability among older adults (SPAOA) plays an important role in enhancing their quality of life and utilization of medical services. This study aimed to evaluate the current state and equity level of SPAOA in Henan Province, China, as well as explore the factors associated with the current state and equity level of SPAOA.

**Methods:**

This study analyzed data from the “2019 Henan Provincial Older Adults Ability Assessment Survey”, which included 4,593 older people (over 60 years old). The relationships among the SPAOA indicators were explored using the Structural Equation Model (SEM). The Dynamic Material Element Analysis (DMA) and Logistic Regression (LR) were used to examine the current state of SPAOA and its associated factors. The equity level of SPAOA and its correlated factors were determined using the concentration index and T Theil index.

**Results:**

SPAOA received an overall score of 91.89 ± 9.83. Daily living, perception ability and mental state were positively correlated with SPAOA (*r* = 0.13, 0.83, 0.11, all *P* < 0.05). Results of LR indicated that the most significant predictors of SPAOA were age, education level, pre-retirement occupation, and income (all *P* < 0.05). The concentration index of SPAOA based on age and income were -0.0058 and 0.0096, respectively. SPAOA had a total T Theil index of 0.030–0.031, and the contribution rate of the difference within the group was greater than 94%.

**Conclusions:**

While the overall level of SPAOA has been demonstrated to be outstanding, persons with a higher educational level and income are likely to benefit the most. The observed unequal distribution of SPAOA is primarily related to disparities in age or income within the group. To better serve older adults and improve their position and equity in terms of social participation ability, policymakers could emphasize older males with little income who live in urban areas, as well as unhealthy older females who live in rural residences.

## Introduction

An aging society is defined as one in which the proportion of the population over 60 years old exceeds 10% or the proportion of the population over 65 years old exceeds 7% [1]. In 2000, China officially became an aging society and it is projected that the proportion of older adults will exceed 25% by the year 2050 [2]. This may be because the average life expectancy in China is close to 77 years, implying that roughly 20% of a person’s life is spent in old age [3]. Henan Province is a large agricultural and newly industrialized province with a population of over 100 million people. In 2019, the older adults over the age of 60 constituted 11.2% of the population, while the ratio of young to old stood at 52.5%, and the older adults dependency coefficient has reached 16.5% [4]. The aging of the population has brought huge challenges to individuals and society, including the rising needs of health, pension and care [5]. In 2002, the World Health Organization proposed the "Active Aging Policy Framework" which changed the traditional aging crisis theory, emphasizing on treating older adults with a positive attitude and highlighting the significance of older adults’ social participation to individuals and society at large [6].

Social participation is a vital component of the older adults’ daily lives, and it is one of the three pillars of the World Health Organization’s (WHO) policy on active aging [7]. Encouraging older adults to participate in social activities can help mitigate the adverse impacts of aging [8]. Increased social participation among older individuals has been shown to reduce the incidence of functional disability, psychological challenges, and the risk of death by more than 15% [9]. The "National Medium and Long-term Plans for Active Response to Population Aging" issued by the State Council of China in 2019 clarified and concertized the strategic goals of actively responding to aging, and formulated comprehensive guidelines for the years 2022, 2035 and the mid-twenty-first century [10]. Zhao YN et al. [11] thought that the older adults’ social involvement in China was rather high, whereas a study conducted by Yu C et al. in Southwest China [12] discovered that their participation had a substantial impact on their quality of life.

The social participation status of older adults mainly depends on their willingness and ability to participate in social activities [13]. The social participation ability among older adults (SPAOA) can be manifested through working ability, time/space/character positioning and social interaction ability [14]. A study by Shi XQ and colleagues conducted in rural areas of Henan province insisted that SPAOA can be associated with a number of internal factors, while Corneliusson L et al. stressed that the primary factor linked to SPAOA is one’s ability to care for oneself in daily life [15]. Not only can older adults obtain comfort and care, but they can also meet their autonomy or ability requirements through family activities [16]. Literature indicates that social participation and cognitive function are inextricably linked, and that there may be a mediation between the two [17]. In 2004, Fratiglioni L et al. [18] proposed that social participation could help mitigate the adverse impacts of stress on cognitive function by influencing and regulating the near-end stress response of cognitive function. Previous animal experimental models have demonstrated that social isolation can result in stress-induced hippocampal atrophy, which may be related to the association between social participation and cognitive function in humans [19]. Mental health has also been demonstrated to be associated with the capacity and willingness of SPAOA [20], as well as engagement in productive activities and volunteer services, all of which serve to prevent depression [21].

Additionally, numerous studies have demonstrated that the ability of older adults to participate actively in social activities is also dependent on mixed factors such as age, sex, area of residence, pre-retirement occupation, education level, and level of income [13]. In China, Nasrin H et al. discovered that the older adults aged between 62–68 years had the highest rate of social participation and that old females had a lower overall level of SPAOA compared to that of males [22, 23]. Yang Fan et al. discovered inequalities in the rate and degree of participation among older adults in urban and rural areas [3] whereas Terraneo M [24] and Bukov A et al. [25] reported that older people with higher education and occupational levels have more resources, such as cognition, communication, and interpersonal relationships. At the same time, adequate medical coverage and community assistance were found to contribute to older adults’ ability to engage in society [22, 26].

Apart from the two binary variables of gender and residency, SPAOA exhibits a declining or growing tendency in response to changes in a variety of categories such as age, educational level, and income. However, such changes are often non-linear and are associated with individual differences [27–29]. For example, in rural China, the scope and quality of social activities of the 65 to 80-year-olds showed a gradual decline, and most of the social activities of the older adults were shown to have stopped after the age of 80 [29]. Nasrin H et al. reported that older adults with a bachelor’s degree or higher had significantly greater social participation ratings than those with a lower educational level [27]. A study has also suggested that health care should be offered equally to older adults of all ages, educational levels, and socioeconomic status, on the basis of prioritizing those in greatest need [30]. As the number and average life expectancy of the older adults continue to rise, it is critical to improve the equity of SPAOA distribution among groups with varying characteristics in order to effectively manage limited resources [25].

In summary, there are several studies on the social participation of older adults, but the majority of these studies focus on the participation status, willingness, and subjective investigation of SPAOA. Few researches have been conducted to examine the equity of SPAOA, its associated factors, or the relationship between the associated factors. For that matter, the novelty of this study compared to the existing studies is that it evaluates the status and equity level of SPAOA objectively and statistically, as well as discusses the elements that correlate the status and equity of SPAOA and the interaction between the correlating factors.

To aid in the creation of effective older adults’ care strategies and to increase the efficiency of older adults’ care services, this study used Structural Equation Model (SEM), Dynamic Material Element Analysis (DMA) method and Logistics Regression (LR) model to explore the constituent elements, current state and correlating factors of SPAOA. The Concentration Index and T Theil index were used to analyze the equity level of SPAOA and the main driving factors leading to inequality.

## Materials and methods

### Data sources

Data on the older population were extracted from the “2019 Henan Provincial Older Adults Ability Assessment Survey”, a cross-sectional survey conducted on the evaluation of the abilities of older adults carried out from January to September 2019. The survey selected participants aged 60 and above residing in Henan province using a multi-stage stratified cluster random sampling technique. In the first stage, 18 province-administered cities in Henan province served as primary sampling units. In the second stage, the stratified sampling method was used to sample urban areas and towns according to the economic level of each province at a ratio of 4:6. The third stage involved layering the sample population according to the proportion of older adults in the three age groups of 60, 70, and 80 in Henan Province (5.0:3.5:1.5), followed by conducting a sample survey. To determine inclusion, the following criteria were used: (1) a minimum age of 60 years; (2) voluntary participation in the survey; and (3) a registered permanent residence in Henan Province for a period of more than six months.

**Fig. 1:**
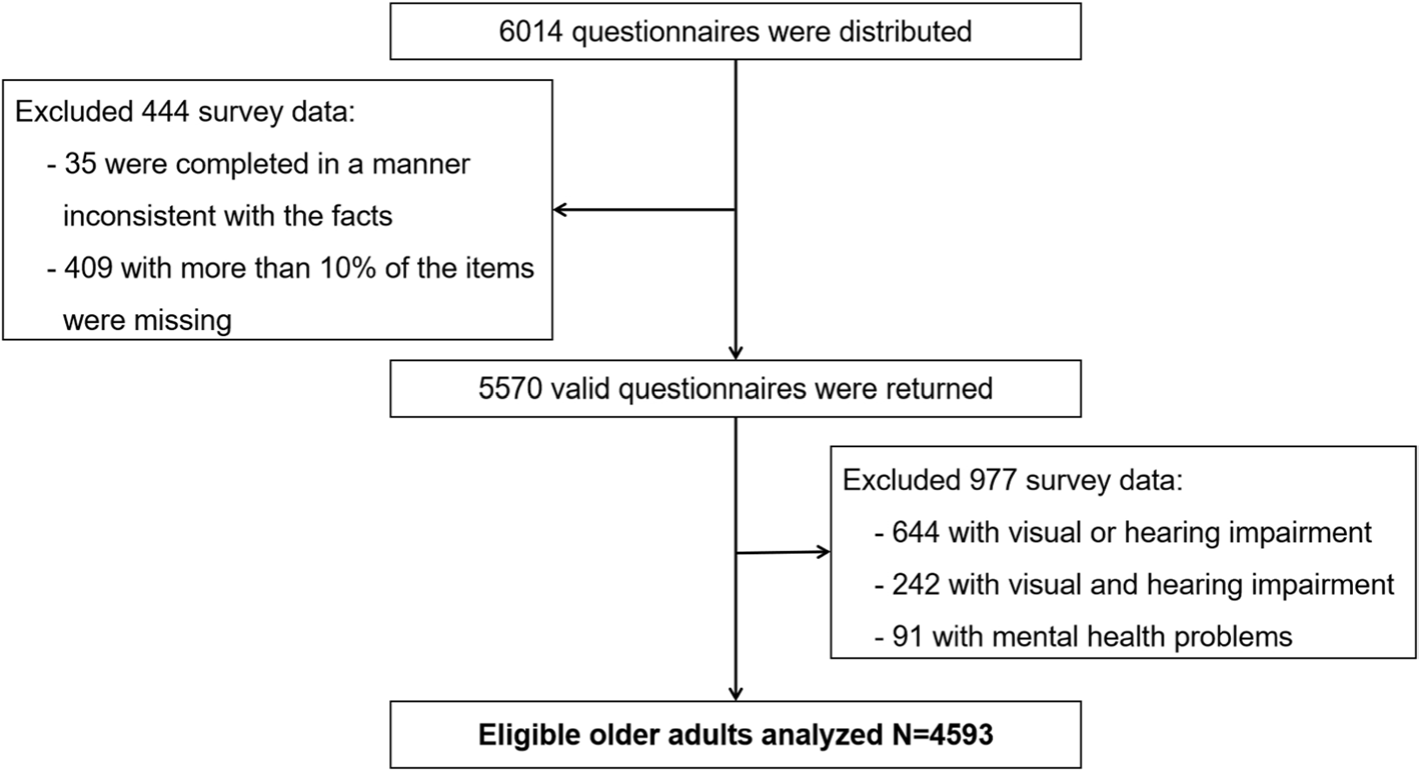
Schematic diagram for sample size estimation

The survey was undertaken by a group of postgraduates majoring in public health. All interviewers received expert training prior to conducting the formal survey. After survey respondents were informed of the survey and agreed to participate, questionnaires were required to be completed and recalled on the spot. For the older adults who were unable to write, the investigators completed the questionnaires on their behalf following extensive interviews. Following the collection of the questionnaires, a telephone follow-up interview was conducted with 20% of the questionnaires. When questionnaires were completed in a manner inconsistent with the facts or when more than 10% of the items were missing, they were deemed unqualified questionnaires. All information was presented in the form of structured questionnaires and collected through face-to-face interviews. As assessed by the Bioethics Committee of Sanquan College of Xinxiang Medical University, the content and procedures of this study met the ethical requirements of international and national biomedical research, and did not involve human or animal experiments, hence it was exempted from formal review procedures. Cross review and repeated input were completed on the same day to ensure data quality. A total of 6,014 questionnaires were distributed, and 5,570 valid questionnaires were returned. After excluding survey subjects who were unable to participate in society on a basic level, such as those with visual impairment, hearing impairment, or mental health problems, this study eventually included 4,593 participants (see Fig. 1).

### Evaluation model

This survey used the civil affairs industry standard developed by the People’s Republic of China’s Ministry of Civil Affairs called "Abilities Assessment of the Old (MZ/T039-2013)" [31], which is now the only authoritative standard in China for assessing the ability of older adults. The questionnaire demonstrated a good reliability and validity, with a Cronbach’s alpha of > 0.70, and it had been widely used to assess disability and social participation in older adults [32–34]. The questionnaire was divided into two sections including "general information sheet" and "ability evaluation sheet", and the specific indicators and their associated meanings used in this study are shown in Table 1 [14, 15, 18, 20].

**Table 1:** The meaning and assigned value of indicators

**Variable category**	**First-level indicators**	**Secondary indicators**	**Meaning**	**Assigned value**
Outcome variables	Daily living (Y1)	eating (x 1)	Refers to the process of using tableware to deliver food from the container to the mouth, chewing, and swallowing	0-10^a^
		bathing (x 2)	The whole process from preparing the bathwater to the completion of the bath	0-4^b^
		grooming (x 3)	Refers to washing face, brushing teeth, combing hair, shaving, etc	0–4
		dressing (x 4)	Refers to putting on and taking off clothes, buckling, zipping, putting on and taking off shoes and socks, and tying shoelaces	0–10
		going to the toilet (x 5)	Including going to the toilet, unbuttoning underwear, wiping, tidying up underwear, flushing	0–10
	Perception ability (Y2)	vision (x 6)	The ability to see words and recognizing objects with their eyes (If the older adults wear reading glasses or myopia, the older adults should evaluate with glasses)	0–4
		hearing (x 7)	The ability to hear other people’s speech, TV, telephone, doorbell, et al. (If the older adults usually wear a hearing aid, the older adults should evaluate with the hearing aid)	0–4
		communication ability (x 8)	The ability to express one’s own needs and understand other people’s words, including non-verbal communication skills	0-2^c^
		consciousness level (x 9)	The state of mind, and the ability to be alert to the surrounding environment	0–2
	Mental state (Y3)	cognitive function (x 10)	Perception and memory of things. Application test method: 1. I said three things, please repeat; 2. Clock drawing test: "Please draw a round clock here and mark 10:45 on the clock"; 3. Recall the words: "Now, please tell me, what are the three things I wanted you to remember just now?"	0–2
		aggressive behavior (x 11)	Physical aggressive behaviors (such as hitting/kicking/pushing/biting/grabbing/falling things) and verbal aggressive behaviors (such as swearing, verbal threats, screaming)	0–2
		depressive symptoms (x 12)	Manifestations of abnormal mood, abnormal activity, and suicidal behavior, etc	0–2
Functional independent variables	SPAOA^d^ (Y4)	Working ability (x 13)	Memory of the original skilled mental work or physical skill work	0–4
		Time/space positioning (x 14)	Perception of time concept and geographic name and location	0–4
		Character positioning (x 15)	Can you know the names and relationships with family and relatives and can you distinguish between acquaintances and strangers	0–4
		Social communication ability (x 16)	The ability to participate in society, adapt to the social environment, and deal with others	0–4

^a^0 means that the older adults need great help or completely rely on others when completing the related behavior, 5 points indicate that the older adults partially need the help of others when completing the related behavior, and 10 indicate that the older adults can complete the related behavior independently

^b^0 means the function or ability of older adults are impaired or very low, 4 means the function or ability of the older adults is normal

^c^0 means behavior of older adults is severe, 2 means no related behavior. The higher the score, the better the ability/function/behavior of older adults

^d^SPAOA: the social participation ability among older adults

#### Structural equation model (SEM)

**Fig. 2:**
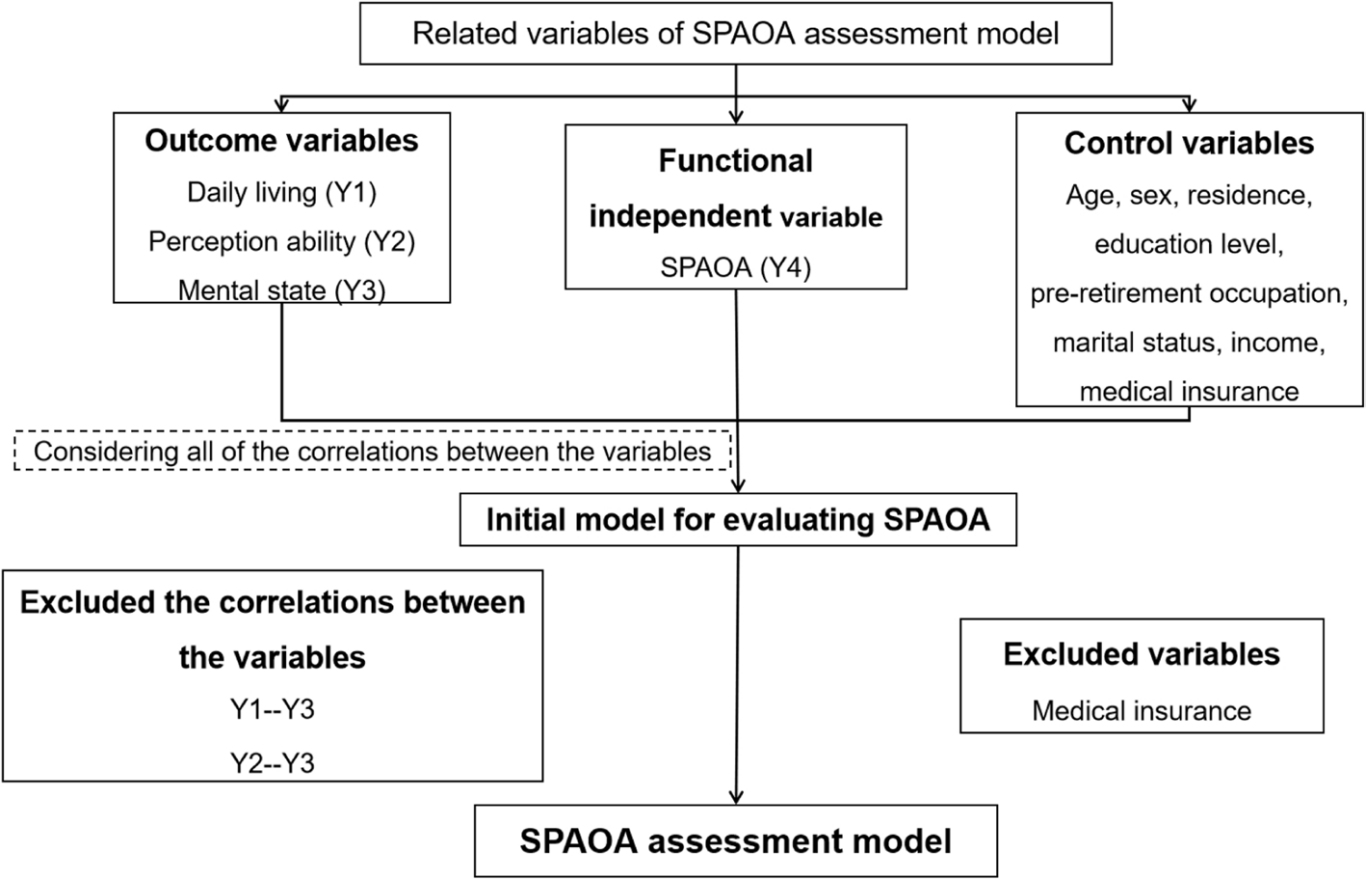
Schematic diagram of variable selection. SPAOA: Social participation ability among older adults

**Fig. 3:**
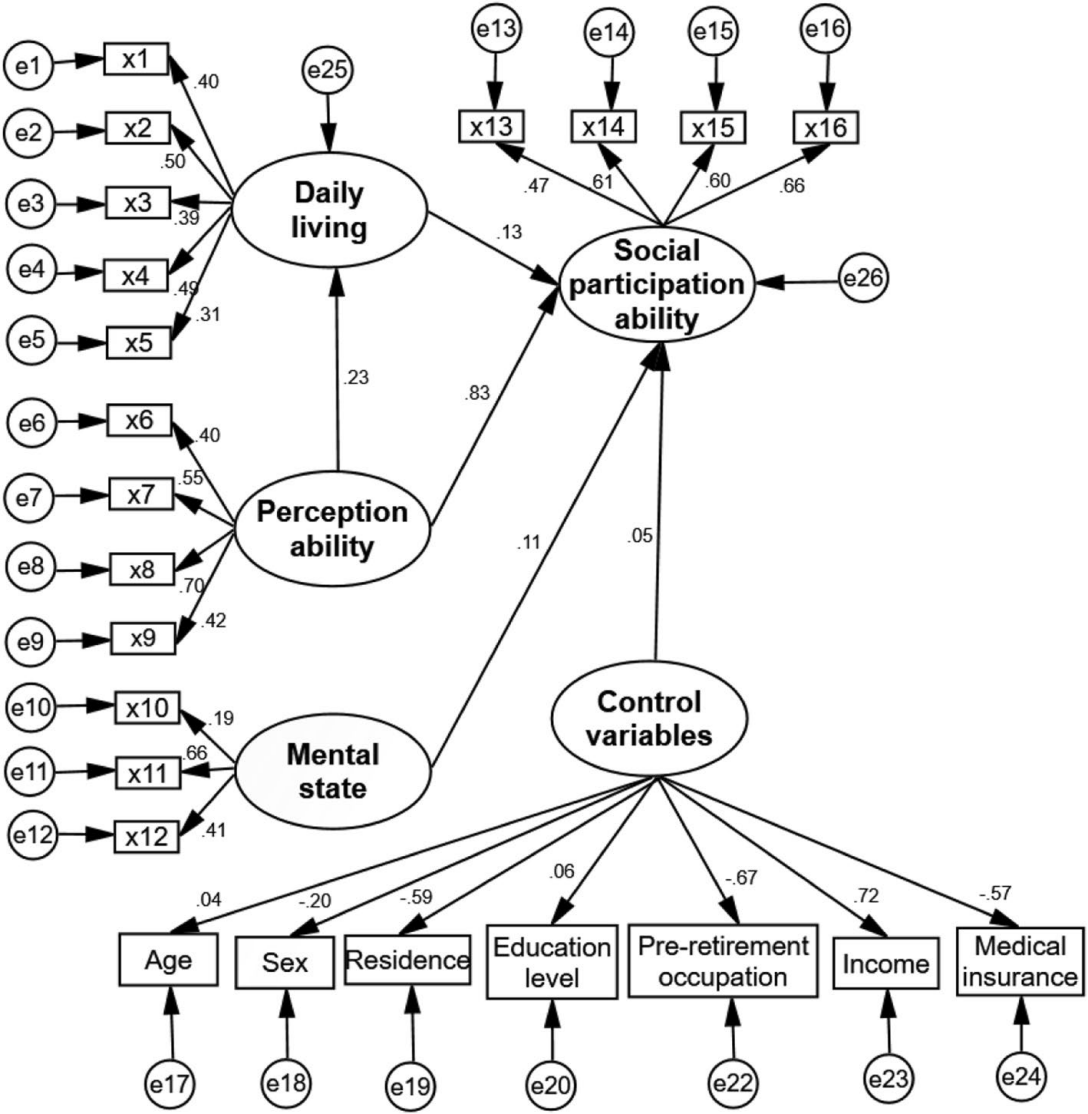
Evaluation model of the social participation ability among older adults

SEM is a statistical method that analyzes the relationship between variables based on the covariance matrix, and it is often used to verify, evaluate and modify the model [35, 36]. In this study, the SEM was used to analyze the relationship between the evaluation indicators of SPAOA. On the basis of field survey data on the older adults’ ability, an initial model for evaluating SPAOA was constructed, which included all of the correlations among the indicators. Additionally, the model included control variables such as age, gender, residence, education level, pre-retirement occupation, marital status, income, and medical insurance [3, 13, 23–29]. According to the test results of the relationship among the indicators, the marital status showed statistical insignificance (*P* > 0.05) and hence was deleted (Fig. 2). The corrected model of SPAOA and the correlation coefficient (r) between the indicators in the model are shown in Fig. 3.

The fitting index of SPAOA assessment model is 1.93, which is less than 2 and close to 1. The sample covariance matrix is similar to the estimated covariance matrix. The model reveals that the approximate root means square error = 0.053, and the goodness of fit index, adjusted goodness of fit index, incremental fit index, standard fit index, and comparative fit index are all greater than 0.80, indicating that the fitting effect of the model is acceptable, but the overall effect is not so notable. This situation may be explained by the huge number of control variables, which may have reduced the goodness of fit of the final model.

### Dynamic matter-element analysis (DMA)

DMA is an objective evaluation method that compares the evaluation results with the theoretical optimal values [37]. The dynamic weight can be specific to each secondary evaluation index with the reward and penalty function which reduces the effect of extreme values by assigning larger values to smaller weights [38]. Because each indicator reflects a dimension of matter, we were able to generate a 12-dimensional matter element *R* (× 1—× 12) using the findings of the SEM analysis for SPAOA evaluation. The value of the reward and penalty functions (d_i_) is given by:

$${d}_{ij}=\frac{1}{{x}_{ij}}$$

where *x*_*ij*_ (*i* = 1, 2, …, 12; *j* = 1, 2, …, 4593) is the evaluation value of each individual indicator. The weight value of each indicator (*W*_*ij*_*)*:

$$W_{ij}=\frac{d_{ij}}{{\sum_1^{12}}d_{ij}}$$

With reference to the relative optimization criterion stating "the bigger the better", the maximum value of the survey results of each index was selected to construct a new matter element among the 12-dimensional complex matter elements of 4,593 older adults (*R*_*0*_). The correlation coefficient of the *i*_*th*_ evaluation index between the *j*_*th*_ older adult and *R*_*0*_ is:

$${L}_{ij}=\frac{{\Delta }_{\mathrm{min}}+{\rho \Delta }_{\mathrm{max}}}{{\Delta }_{ij}+{\rho \Delta }_{\mathrm{max}}}$$

where: *Δ*_*ij*_ is the absolute difference between the value of the *i*_*th*_ evaluation index of the best social participation ability and the corresponding index value of the *j*_*th*_ older adult (*i* = 1, 2, …, 12; *j* = 1, 2, …, 4593); *Δ*_*min*_ is the minimum value of the absolute difference of the index value *Δ*_*ij*_; *Δ*_*max*_ is the maximum value of the absolute difference of the index value *Δ*_*ij*_; *ρ* is the resolution coefficient, which is 0.5 generally.

A comprehensive measurement of the correlation between the social participation ability among each older adult and the best social participation ability is *L*_*0j*_ (SAPOA evaluation result, 0–100):

$$L_{0j}=100\frac{{\sum_{j=1}^{4593}}{\sum_{i=1}^{12}}W_{ij}L_{ij}}{4593}$$

#### Concentration index and T Theil index

As one of the equity measurement methods, the concentration index is commonly used to measure the equity of income, health and sanitation services [39]. The commonly used calculation methods for the concentration index are the geometric method and the covariance method. Fu XZ et al. [40] used the covariance method to calculate the concentration index of older adults health service utilization while Wagstaff A et al. [41] applied geometric methods to calculate the concentration index of the malnutrition status of Vietnamese people. Considering that SPAOA variable in the current study is numerical, we used the geometric method to calculate the concentration index (G) of SPAOA:

$$\mathrm{G}=1-\sum_{i=0}^{4592}\left({x}_{i+1}-{x}_{i}\right)\left({y}_{i+1}-{y}_{i}\right)$$

where x_i_ is the cumulative percentage of the number of older adults and y_i_ represents the cumulative percentage of SPAOA. The value of the concentration index ranges between -1 and 1. The concentration index is more equitable when its absolute value is close to 0. A negative concentration index indicates a lower level clustering, while a positive concentration index indicates a higher level clustering [41].

The T Theil index is frequently used to quantify differences in resource allocation within and between groups. The smaller the value of T Theil index the better [42]. The T Theil index can be used to explain the total difference in discrepancies within and across groups, which can help to further reflect the equity of SPAOA and identify the causes of skewness based on the concentration index results [43]. The specific calculation formula is as follows:

$${\mathrm{T}}_{\mathrm{i}}=\sum_{j}\left(\frac{{r}_{ij}}{{r}_{i}}\right)\times \mathrm{log}\left(\frac{{r}_{ij}/{r}_{i}}{{n}_{ij}/{n}_{i}}\right)$$

$${\mathrm{T}}_{\mathrm{Between groups}}=\sum_{i}{R}_{i}\times {T}_{i}$$

$${\mathrm{T}}_{\mathrm{Within groups}}=\sum_{i}{R}_{i}\times \mathrm{log}\left(\frac{{R}_{i}}{{N}_{i}}\right)$$

$${\mathrm{T}}_{\mathrm{Total}}={\mathrm{T}}_{\mathrm{Between groups}}+{\mathrm{T}}_{\mathrm{Within groups}}$$

Among them, T_Total_, T_Between groups_, T_Within groups_, and T_i_ represent the T Theil index of the total, between groups, within group and each older adult, respectively; r_ij_ is the population of each older adult (in this study, 1); r_i_ is the score of SPAOA; n_ij_ is total number of the older adults in each group; n_i_ is the sum of the scores of SPAOA in each group; R_i_ is the proportion of the older population in each group to the total number of the older adults; N_i_ is the proportion of the score of SPAOA in each group to the total score.

### Statistical analysis

This study established the evaluation model of SPAOA by using SEM. DMA was used to assess the current status of SPAOA. LR was used to analyze the correlating factors of SPAOA. The Concentration Index and T Theil index were used to analyze the equity of SPAOA and its main driving factors leading to inequality. Data entry was done by using Epidata 3.0 software. Statistical analysis was carried out using Excel 2019, SPSS 20.0 and Amos 20.0 software.

## Results

### Basic information

The descriptive characteristics of the study participants are listed in Table 2. The majority (51.12%) of study participants were female, while 48.88% were male. Participants aged 60–69 and over 80 accounted for 53.69% and 10.26%, respectively. More than 72% of the older adults lived in rural areas, 62.63% had an education level of elementary school or below, and over 24% were unemployed before retirement. More than 53% of the older adults had a monthly income of 1,000 Chinese yuan or less, and only 8.38% of the older adults had a monthly income of more than 3,000 Chinese yuan. In terms of medical insurance, less than 3% had not purchased any insurance. At the same time, the analysis found statistical differences in SPAOA by age, sex, residence, education level, pre-retirement occupation, income and medical insurance (*P* < 0.05).

**Table 2:** Characteristics of the study participants (*N* = 4593)

**Index**	**older adults (person (%))**	**Score**^**a**^** (**$\overline{x} \pm s$***)***	***t/F***	***P***
**Age (years)**				
60 ≤ Age < 70	2466 (53.69)	93.66 ± 8.47	33.663	< 0.001
70 ≤ Age < 80	1656 (36.05)	92.90 ± 8.98		
Age > = 80	471 (10.26)	89.99 ± 10.79		
**Sex**				
Male	2348 (51.12)	93.51 ± 8.86	3.870	< 0.001
Female	2245 (48.88)	92.49 ± 9.07		
**Residence**				
Urban	1249 (27.19)	94.40 ± 8.43	6.681	< 0.001
Rural	3344 (72.81)	92.49 ± 9.12		
**Educational level**				
Illiteracy	1204 (26.21)	90.07 ± 9.77	66.656	< 0.001
Primary school	1673 (36.42)	92.84 ± 9.12		
Junior high school	1031 (22.45)	94.61 ± 7.78		
High School/Technical School	538 (11.71)	95.77 ± 7.45		
College degree and above	147 (3.21)	97.63 ± 5.18		
**Pre-retirement occupation**^b^				
Government staffs	799 (17.40)	95.19 ± 8.07	31.063	< 0.001
Industry and service workers	748 (16.29)	94.12 ± 8.43		
Agricultural staffs	1920 (41.80)	91.86 ± 9.29		
Others	1126 (24.51)	92.68 ± 9.05		
**Income (Chinese Yuan/month)**				
1000 and below	2452 (53.39)	92.16 ± 8.93	22.687	< 0.001
1001–2000	1147 (24.97)	93.28 ± 9.43		
2001–3000	609 (13.26)	94.31 ± 8.63		
3001 and above	385 (8.38)	95.56 ± 7.56		
**Medical insurance**^c^				
Basic Medical Insurance for Urban Employees^d^	619 (13.48)	95.10 ± 8.37	21.665	< 0.001
Basic Medical Insurance for Urban Residents^e^	663 (14.44)	94.27 ± 8.24		
New Rural Cooperative Medical System^f^	3156 (68.71)	92.39 ± 9.11		
Others	155 (3.37)	91.89 ± 9.83		

^a^Score: The score of the social participation ability among older adults (SPAOA) (0–100)

^b^Pre-retirement occupation: Occupations that older people mainly engaged in before retirement

^c^Medical insurance: The main type of medical insurance that older adults possess

^d^Basic Medical Insurance for Urban Employees: In China, it refers to basic medical insurance program mandated by law, in which all urban firms’ employees must enroll. The insurance premium shall be borne by both the employer and the employee

^e^Basic Medical Insurance for Urban Residents: It is a kind of basic medical insurance for urban residents. Insurance premiums are mainly paid by individual residents (families), supplemented by appropriate government subsidies. ^f^New Rural Cooperative Medical System: It is a kind of basic medical insurance established for rural residents, which raises funds by means of individual payment, collective support and government funding

### The current state of SPAOA

**Fig. 4:**
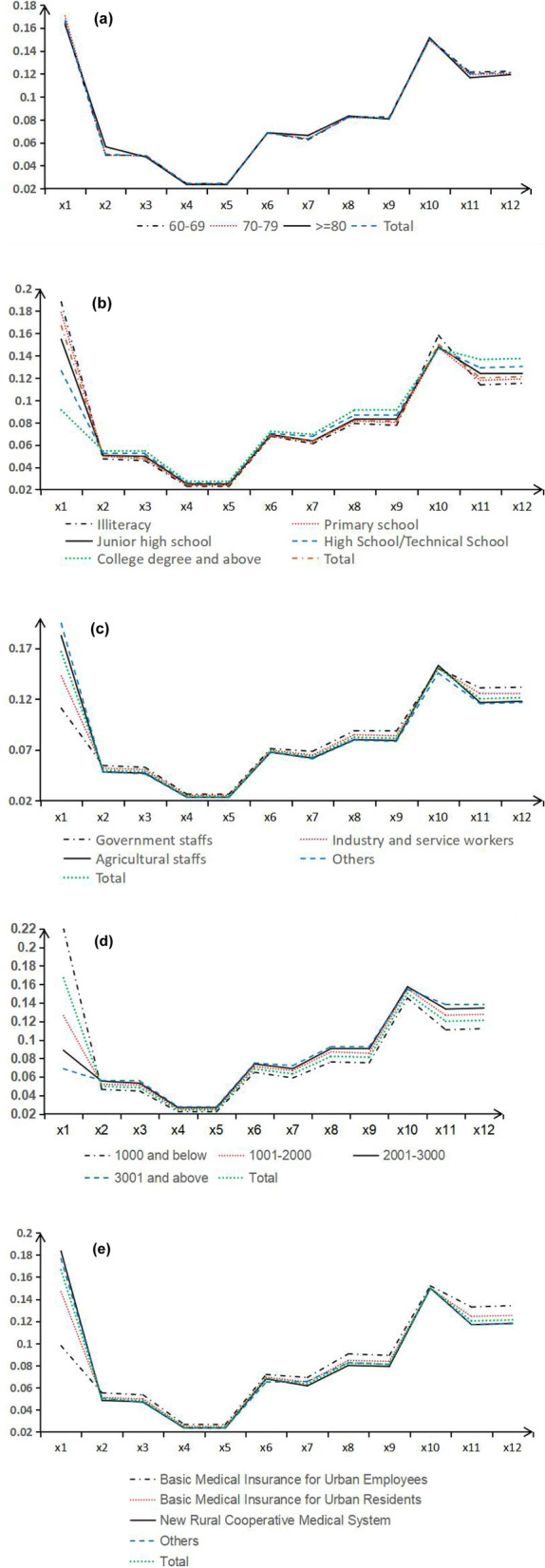
The weight of evaluation index of the social participation ability among older adults. **a** Age; **b** Education level; **c** Pre-retirement occupations; **d** Income; **e** Medical insurance

The survey data was substituted into the SEM constructed by this study, and applied the DMA method for analysis. The results showed that the total score of SPAOA was 91.89 ± 9.83 (0–100). For each evaluation dimension, the weight coefficients for eating, cognitive function, and depressive symptoms were relatively large, at 0.1664, 0.1502, and 0.1212, respectively. The three dimensions with the highest scores included eating, aggressive behavior, and depressive symptoms, with the scores 16.61 ± 3.22, 11.95 ± 1.41, 11.92 ± 1.51 (Table 3). The study also analyzed the dynamic changes in the weights of SPAOA evaluation indicators by age, education level, pre-retirement occupation, income and medical insurance, as shown in Fig. 4.

**Table 3:** The social participation ability among older adults evaluation results

**Indicators**	**Weight coefficient**	**Sequence**	**Score**^**a**^ $\left(\overline{{\varvec{x}}}\pm {\varvec{s} }\right)$	**Sequence**
**Daily living**				
Eating	0.1664	1	16.61 ± 3.22	1
Bathing	0.0499	9	4.82 ± 0.55	9
Grooming^b^	0.0484	10	4.80 ± 0.55	10
Dressing^c^	0.0242	11	2.39 ± 0.29	12
Going to the toilet	0.0240	12	2.38 ± 0.28	11
**Perception ability**				
Vision^d^	0.0686	7	5.54 ± 0.99	8
Hearing	0.0633	8	5.84 ± 0.86	7
Communication ability	0.0822	5	7.90 ± 1.07	6
Consciousness level^e^	0.0813	6	7.91 ± 1.03	5
**Mental state**				
Cognitive function^f^	0.1502	2	10.95 ± 2.65	4
Aggressive behavior^g^	0.1202	4	11.95 ± 1.41	2
Depressive symptoms^h^	0.1212	3	11.92 ± 1.51	3
**Total**	1		93.01 ± 8.98	

^a^Score: The score of the social participation ability among older adults (SPAOA)

^b^Grooming: Refers to washing face, brushing teeth, combing hair, shaving, etc.

^c^Dressing: Refers to putting on and taking off clothes, buckling, zipping, putting on and taking off shoes and socks, and tying shoelaces

^d^Vision: The ability of seeing words and recognizing objects with one’s eyes

^e^Consciousness level: The state of mind, and the ability to be alert to the surrounding environment

^f^Cognitive function: Perception and ability to memorize

^g^Aggressive behavior: Physical aggressive behaviors and verbal aggressive behaviors

^h^Depressive symptoms: Manifestations of abnormal mood, abnormal activity, and suicidal behavior

### Analysis on the associated factors of SPAOA based on LR

The LR method was used to analyze the associated factors linked with SPAOA. According to univariate analysis, the existing research results of social participation ability [44] and avoiding false negatives; the age, sex, residence, education level, pre-retirement occupation, income and medical insurance were included as independent variables into the LR model. In this study, the SPAOA score less than 90 points was assigned a value of 0, and the score greater than or equal to 90 points was assigned a value of 1. The values assignment to the independent variables was shown in Table 4. The tolerance (Tol) of the regression model was greater than 0.53, and the variance inflation factor (VIF) was less than 1.88, indicating that the collinearity was at an acceptable level.

**Table 4:** Variables assignment

**Variables**	**Assignment**
SPAOA^a^	< 90 points = 0, ≥ 90 points = 1
Age	60 ≤ Age < 70 = 1, 70 ≤ Age < 80 = 2, Age > = 80 = 3
Sex	Male = 0, Female = 1
Residence	Urban = 0, Rural = 1
Educational level	Illiteracy = 1, Primary school = 2, Junior high school = 3, High School/Technical School = 4, College degree and above = 5
Pre-retirement occupation^b^	Government staffs = 1, Industry and service workers = 2, Agricultural staffs = 3, Others = 4
Income	1000and below = 1, 1001–2000 = 2, 2001–3000 = 3, 3001and above = 4
Medical insurance^c^	Basic Medical Insurance for Urban Employees = 1, Basic Medical Insurance for Urban Residents = 2, New Rural Cooperative Medical System = 3, Others = 4

^a^SPAOA: Score of the social participation ability among older adults

^b^Pre-retirement occupation: Occupations in which older individuals spend the majority of their time prior to retirement

^c^Medical insurance: The most common type of medical insurance held by older adults

Regression analysis results showed that SPAOA was mainly correlated with age, education level, pre-retirement occupation and income (*P* < 0.05). Among them, the social participation ability of the 60–70 and 70–80 older adults were 1.711 and 1.521 times that of the older adults over 80 (*OR* = 1.711, *95%CI* = 1.380 ~ 2.120; *OR* = 1.521, *95%CI* = 1.221 ~ 1.895). The social participation ability of the older adults with illiteracy, primary school and junior high school education level were 0.117, 0.321, and 0.408 times that of the older adults with college education, respectively (*OR* = 0.177, *95%C*I = 0.098 ~ 0.319; *OR* = 0.321, *95%CI* = 0.179 ~ 0.567; *OR* = 0.408, *95%CI* = 0.228 ~ 0.731). The social participation ability of the older adults engaged in agriculture was 0.695 times that of the unemployed before retirement (*OR* = 0.695, *95%CI* = 0.592 ~ 0.817). The correlate of sex, residence and medical insurance on SPAOA were not statistically significant (*P* > 0.05). See Table 5.

**Table 5:** Logistic regression (LR) results

**Indicators**	***β***^***a***^	***SE***^***b***^	***Wals***^***c***^	***P***	***OR (95%CI)***
**Age (> = 80 years)**^d^	-	-	24.047	< 0.001	-
60 ≤ Age < 70	0.537	0.110	23.999	< 0.001	1.711(1.380 ~ 2.120)
70 ≤ Age < 80	0.419	0.112	13.981	< 0.001	1.521(1.221 ~ 1.895)
**Sex (Female)**	-0.02	0.069	0.081	0.776	0.981(0.856 ~ 1.123)
**Residence (Rural)**	0.039	0.100	0.148	0.701	1.039(0.854 ~ 1.265)
**Educational level (College degree and above)**	-	-	120.519	< 0.001	-
Illiteracy	-1.733	0.302	33.045	< 0.001	0.177(0.098 ~ 0.319)
Primary school	-1.137	0.298	14.523	< 0.001	0.321(0.179 ~ 0.576)
Junior high school	-0.897	0.298	9.088	0.003	0.408(0.228 ~ 0.731)
High School/Technical School	-0.527	0.302	3.042	0.081	0.590(0.326 ~ 1.067)
**Pre-retirement occupation**^e^ **(Others)**			23.877	< 0.001	-
Government staffs	-0.052	0.134	0.149	0.699	0.950(0.73 ~ 1.235)
Industry and service workers	-0.079	0.114	0.48	0.489	0.924(0.739 ~ 1.155)
Agricultural staffs	-0.363	0.082	19.634	< 0.001	0.695(0.592 ~ 0.817)
**Income (3001and above)**	-	-	8.035	0.045	-
1000 and below	-0.262	0.167	2.457	0.117	0.770(0.555 ~ 1.068)
1001–2000	-0.062	0.166	0.138	0.710	0.940(0.679 ~ 1.302)
2001–3000	-0.015	0.172	0.007	0.932	0.985(0.703 ~ 1.381)
**Medical insurance (Others)**	-	-	4.341	0.227	-
Basic Medical Insurance for Urban Employees^f^	0.286	0.214	1.784	0.182	1.331(0.875 ~ 2.026)
Basic Medical Insurance for Urban Residents^g^	0.382	0.197	3.788	0.049	1.466(0.996 ~ 2.158)
New Rural Cooperative Medical System^h^	0.201	0.176	1.315	0.252	1.223(0.867 ~ 1.726)
**Constant**	1.566	0.406	14.911	< 0.001	4.788

^a^β: β refers to the regression coefficient

^b^SE: SE refers to standard error

^c^Wals: Wals is a statistic used to test whether the independent variable has an effect on the dependent variable

^d^: Indicates comparative items

^e^Pre-retirement occupation: Occupations in which older individuals spend the majority of their time prior to retirement

^f^Basic Medical Insurance for Urban Employees: In China, it refers to basic medical insurance program mandated by law, in which all urban firms’ employees must enroll. The insurance premium shall be borne by both the employer and the employee

^g^Basic Medical Insurance for Urban Residents: It is a kind of basic medical insurance for urban residents. Insurance premiums are mainly paid by individual residents (families), supplemented by appropriate government subsidies

^h^New Rural Cooperative Medical System: It is a kind of basic medical insurance established for rural residents, which raises funds by means of individual payment, collective support and government funding

### Current state and the associated factors of the equity of SPAOA

#### Current state of the equity

This study sorted the age, education level, and income from low to high, and sorted the pre-retirement occupations by government workers, industry and service industries, agriculture, and unemployment status. It was calculated that the concentration index of SPAOA based on education level and income was 0.0154 and 0.0096, respectively, that was the evaluation of SPAOA tends to older adults with higher education level and income. The allocation of SPAOA between age and pre-retirement occupation were basically equal (concentration index = -0.0058, -0.0093). Simultaneously, the results of the analysis by gender and place of residence indicated that, in terms of age and education level, older females had a higher absolute value of the concentration index of social participation ability than older males, and rural older adults had a higher absolute value than urban older adults. See Table 6.

**Table 6:** Concentration Index of the social participation ability among older adults

**Indicators**	**Total**	**Sex**		**Residence**	
		**Male**	**Female**	**Urban**	**Rural**
Age	-0.0058	-0.0049	-0.0070	-0.0057	-0.0063
Educational level	0.0154	0.0135	0.0161	0.0139	0.0140
Pre-retirement occupation^a^	-0.0093	-0.0066	-0.0067	-0.0088	-0.0038
Income	0.0096	0.0050	0.0034	0.0042	0.0041

^a^Pre-retirement occupation: Occupations in which older individuals spend the majority of their time prior to retirement

#### Factors correlating equity

The total T Theil index of SPAOA was between 0.030 and 0.031. The contribution rate of the intra-group difference was greater than the contribution rate of the inter-group difference which exceeded 94%, indicating that the difference in the equity of the SPAOA was mainly due to the difference between groups. This means that the total T Theil index of older females was larger than that of older males, and that the total T Theil index of older adults from rural areas was greater than that of older adults residing in urban locality. Except for income and age, the contribution rate of the intra-group T Theil index of social participation ability among older males in rural residence was greater than the total level while the contribution rate of the intra-group T Theil index of social participation ability among older females residing in urban areas was less than the total level. See Table 7.

**Table 7:** T Theil Index and contribution rate (%) of the social participation ability among older adults

**Indicators**	**T Theil index (contribution rate)**	**Total (10**^**–2**^**)**	**Sex**		**Residence**	
			**Male**	**Female**	**Urban**	**Rural**
Age	Total T Theil Index	3.0489	2.8821	3.1984	2.4824	3.2146
	T Theil index within group (%)	3.0082 (98.66)	2.8554 (99.07)	3.1375 (98.10)	2.4379 (98.21)	3.1697 (98.60)
	T Theil index between groups (%)	0.0408 (1.34)	0.0267 (0.93)	0.0608 (1.90)	0.0445 (0.79)	0.0449 (1.40)
Educational level	T Total Theil Index	3.0489	2.8821	3.1984	2.4824	3.2146
	T Theil index within group (%)	2.8774 (94.37)	2.7507 (95.44)	3.0063 (93.99)	2.3303 (93.87)	3.0733 (95.61)
	T Theil index between groups (%)	0.1716 (5.63)	0.1314 (4.56)	0.1921 (6.01)	0.1521 (6.13)	0.1412 (4.39)
Pre-retirement occupation^a^	T Total Theil Index	3.0401	2.8821	3.1984	2.4824	3.2146
	T Theil index within group (%)	2.9719 (97.76)	2.8366 (98.42)	3.1131 (97.33)	2.4222 (97.58)	3.1786 (98.88)
	T Theil index between groups (%)	0.0682 (2.24)	0.0454 (1.58)	0.0852 (2.67)	0.0602 (2.42)	0.0360 (1.12)
Income	T Total Theil Index	3.1074	2.8821	3.1984	2.4824	3.2146
	T Theil index within group (%)	3.0345 (98.31)	2.8607 (98.20)	3.1870 (98.20)	2.4628 (98.29)	3.1981 (99.58)
	T Theil index between groups (%)	0.0729 (1.69)	0.0214 (1.80)	0.0114 (1.80)	0.0196 (1.71)	0.0164 (0.42)

^a^Pre-retirement occupation: Occupations in which older individuals spend the majority of their time prior to retirement

## Discussion

The purpose of this study was to assess the current state and equity level of SPAOA as well as to investigate the factors correlating with it through an analysis of data from a survey on the abilities of older adults in Henan Province, China. The findings show that the overall level of SPAOA was sufficient but mainly associated with age, education level, pre-retirement occupation and income. The equity of the SPAOA distribution based on the Gini coefficient was acceptable, and the skewness was caused mostly by differences within the group based on age or income.

Specifically, the overall score of SPAOA in Henan Province of China was found to be 91.89 ± 9.83, and the daily lifestyle, perception ability, mental state were positively correlated with SPAOA. The SME results indicate that the perception ability is considered to be the most relevant factor for SPAOA. The correlation between daily life, mental state and SPAOA is relatively low, which is consistent with other studies [45]. On the other hand, both daily life and mental state have a direct impact on SPAOA. While perceptive ability has a direct effect on SPAOA, this study discovered that it also has an indirect effect on SPAOA in daily life. At the same time, consistent with the SEM analysis, the DMA results based on the extended set theory indicate that the mental state occupies the relatively largest weight. The scores and importance of "eating behavior" and "depression symptoms" dimensions are relatively high, as is the importance of cognitive function and aggressive behavior. The older adults are very concerned about factors that directly and significantly correlate with their health, such as their own diet and mental conditions. However, the older adults’ level of daily living activities and perception is low, and their level of attention needs to be enhanced further.

The results also showed that SPAOA declines with age. The social participation ability of 60–70 and 70–80 years older adults are 1.711 and 1.521 times that of the older adults over 80, respectively. On the other hand, while older adults’ educational attainment benefits SPAOA, the effect reduces as educational attainment reaches high school/technical school. Similar findings were shown by a study conducted by Astrid E et al. [46] whereas SPAOA increased to a considerable extent as educational level increased, but as educational level continues to grow, SPAOA essentially was shown to remain steady [46]. This may be related to the fact that in China, students study fundamental knowledge about life and society in high school/technical school and below, whereas college education focuses on professional knowledge [47]. The current study also found that the older adults who engaged in agricultural activities have relatively the lowest social participation ability. One probable explanation is that, on average, farmers in developing countries have a low economic and political status.

In addition, this study found that SPAOA was generally fair, but its beneficiaries were more likely to be persons with advanced education and income. In the field of education, this trend was more pronounced among older females who reside in rural areas and it was more pronounced among older males living in urban areas in terms of income. The finding is similar to the research by Gallo HB et al. which established a positive correlation between income and ability to participate in social activities [48]. One possible explanation is that the older adults in China’s rural areas have limited access to education and income due to the country’s economic and educational constraints. In addition, the distribution of SPAOA across age and pre-retirement occupations was found to be skewed. This could be because the survey subjects are predominantly government employees, workers, and farmers, and pre-retirement occupation has a significant impact on the economic status of older adults. Given that age is an unmodifiable characteristic, policymakers and organizers should prioritize the social participation of rural older females who face physical, psychological, and social limitations [49].

Finally, the inequality in the distribution of SPAOA was primarily attributable to variances within the group, which reminded that SPAOA was connected with a multitude of parameters concurrently, and no single feature can alone reflect an individual’s total ability [50]. The inequity situation within the population with the same characteristics should receive more attention. Therefore, we suggest that when the government formulates relevant policies and pension institutions to provide services, a greater emphasis should be given to the individual abilities of older adults, rather than simply formulating classification criteria solely based on their age and income.

This study has several advantages: Firstly, the authors focused our research direction on SPAOA. Secondly, the study used SEM to explain the association between a number of confounding variables and SPAOA. Thirdly, the evaluation results of SPAOA based on the DMA model and concentration index can provide evidence-based reference for decision-makers engaged in the practice of older adults’ service security reform. However, a notable limitation of the study is that due to the limited sample coverage and disparities in economic and social features among the participants, hence the findings of this study may not be fully applicable or generalized to other regions. Additionally, the three methodologies used in this study, the DMA, the Concentration index, and the T Theil index, have stringent requirements for the quantity and quality of data, limiting their applicability to other similar investigations. Thirdly, given the cross-sectional nature of the current study, the authors believe that there may be other important confounding factors, such as intimate relationships and emotional status of the survey subjects that deserve future exploration and analysis.

## Conclusions

This study evaluates the current state and equity level of SPAOA in Henan Province, China, as well as exploring the factors that are associate with social participation ability and equity. The results showed that the overall level of SPAOA is quite high, and that it is mainly associated with age, education level, pre-retirement occupation and income. At the same time, the low fairness of the SPAOA distribution is mainly due to differences within the group. Therefore, the authors suggest that policymakers could focus on low-income older males in urban regions as well as unhealthy older females in rural areas in order to better serve the older population and improve the status and equity of SPAOA. On the other hand, rather than focusing exclusively on increasing resources to address the issue of insufficient older adults’ services, policymakers should also consider increasing the level of SPAOA when developing policies regarding the older population.

## Data Availability

The data that support the findings of this study are available from Health Commission of Henan Province but restrictions apply to the availability of these data, which were used under license for the current study, and so are not publicly available. Data are however available from the authors upon reasonable request and with permission of Health Commission of Henan Province.

## Abbreviations

SPAOA: Social participation ability among older adults
SEM: Structural Equation Model
DMA: Dynamic Material Element Analysis
LR: Logistic regression
